# Three More Learning Points

**DOI:** 10.1371/journal.pmed.0020030

**Published:** 2005-01-25

**Authors:** Ignacio Garcia-Doval

**Affiliations:** Complexo Hospitalario de PontevedraPontevedraSpain

After reading the Learning Forum by Fleming and Lynn [1], I would like to suggest three learning points that, in my opinion, should receive more attention.

(1) Morphology: the essential point of dermatological diagnosis is morphology, a low tech, but hard to master, skill. Dermatological diagnosis, as any other medical diagnosis, starts by collecting adequate information from the patient, and follows by its elaboration. Many doctors consider that dermatological diagnosis can be made on a quick recognition basis, but an ordered and syndromic approach is essential to get to an adequate diagnosis. I think that most dermatologists would agree that a good description of a patient by an experienced colleague is a better starting point for diagnosis than many pictures. I would describe the lesions seen in Figure 1 of [1] not simply as shallow ulcers, but as clearly polycyclic erosions (a finding highly suggestive of herpetic infection).

(2) Indicated investigations: Tzanck test is the microscopic evaluation of cell morphology on a cutaneous smear. It can be done in about 15 minutes, requiring a microscope and a trained doctor. Access to this test is probably much easier than to viral cultures or polymerase chain reaction tests. In this setting, a positive Tzanck test would be enough to confirm the clinical diagnosis at a minimum cost. Considering the widespread audience of *PLoS Medicine*, with many readers in less developed countries, this test should not be forgotten.

(3) This case, and the suspicion about systemic manifestations of skin disease, is a wonderful opportunity to disseminate an old concept, very frequently forgotten in medical literature: the skin is an organ, in fact, the biggest one in the body. Its main functions are to act as a barrier, to control temperature, to serve immunological and hormonal roles, and, physiologically less important but very important for patient well-being, to participate in personal relationships. When these functions are not adequately performed, skin failure appears, exactly as is the case with heart or renal failure. Skin failure can have many manifestations, including noninfectious fever, bacteremia, or sepsis. As is the case with renal or cardiac failure, it is easier and more practical to learn about this syndrome than to discuss the systemic manifestations of the many diseases that can cause it. I would highly recommend the following references for doctors interested in the subject: [2,3].
